# Soft tissue changes in the foot of people with diabetes

**DOI:** 10.1186/1757-1146-8-S2-O20

**Published:** 2015-09-22

**Authors:** Belinda Ihaka, Wayne Hing, Keith Rome

**Affiliations:** 1 Faculty of Health & Environmental Sciences, Health & Rehabilitation Research Institute, School of Podiatry, AUT University, Auckland, 1142, New Zealand; 2 Faculty of Health Sciences and Medicine mBond University QLD 4229, Australia

**Keywords:** Diabetes, Plantar fascia thickening, Plantar pressure

## Background

Māori have poorer health outcomes compared to non-Māori and are over-represented in amputation and mortality rates in Aotearoa. There is limited knowledge on the biomechanical parameters of Māori feet with diabetes and peripheral neuropathy. The primary aim was to evaluate differences in plantar fascia thickness and plantar pressures under the forefoot region between Māori and non-Māori with and without diabetes and peripheral neuropathy. A secondary aim was to determine the relationship between plantar fascia thickness and plantar forefoot pressures under the forefoot in Māori with diabetes.

## Methods

A cross-sectional observational study was conducted on 36 participants recruited from two clinical sites (South Auckland and North Shore, Auckland). Participants who met the inclusion criteria were divided into four groups: Māori with diabetes, Māori with no diabetes, non-Māori with diabetes and non-Māori with no diabetes. Plantar fascia thickness was measured by ultrasound. Forefoot peak plantar pressure and pressure time integrals were evaluated using a platform pressure-system. Significant differences were analysed using a Kruskal-Wallis test and a Pearson's *r*-correlation to analyse the relationship between plantar fascia thickness and plantar pressure variables.

## Results

No significant demographic differences were found across the four groups (*p*>0.05). Plantar fascia thickness showed significant differences between the four-groups (*p*=0.02). Post-hoc analysis demonstrated significant increases between Māori with diabetes and non-Māori with no diabetes (*p*=0.04); and non-Māori with diabetes and non-Māori with no diabetes (*p*=0.01). Peak plantar pressure demonstrated significant differences across the groups for the 2^nd^/3^rd^ metatarsophalangeal joints (*p*=0.01) and 4^th^/5^th^ metatarsophalangeal joints (*p*=0.02), but no difference for the 1^st^ metatarsophalangeal joint (*p*=0.10). No significant differences in pressure time integrals were found in the four groups across the forefoot region (*p*>0.05). In Māori with diabetes, a significant strong relationship was found between plantar fascia thickness and peak plantar pressure at the 4^th^/5^th^ metatarsophalangeal joints (r = 0.77; *p* =0.01).

## Conclusion and clinical relevance

The preliminary results found differences to the plantar fascia and increased plantar pressures in the lateral forefoot of Māori with diabetes and peripheral neuropathy. These findings suggest that biomechanical foot characteristics of Maori may differ from other populations and should be considered during clinical evaluation.

